# Wet-Induced Fabrication of Heterogeneous Hump-on-String Fibers

**DOI:** 10.3390/ma8074249

**Published:** 2015-07-13

**Authors:** Cheng Song, Ruofan Du, Yongmei Zheng

**Affiliations:** 1 Key Laboratory of Bio-Inspired Smart Interfacial Science and Technology of Ministry of Education, School of Chemistry and Environment, Beijing University of Aeronautics and Astronautics, Beijing 100191, China; E-Mail: songcheng@buaa.edu.cn; 2 National Laboratory for Computational Fluid Dynamics, School of Aeronautic Science and Engineering, Beijing University of Aeronautics and Astronautics, Beijing 100191, China; E-Mail: duruofan@buaa.edu.cn

**Keywords:** wet-induced assembly, heterogeneous, hump-on-string fiber

## Abstract

Inspired by the high adhesiveness of the electrospun fiber, we propose a method to fabricate multi-scale heterogeneous hump-on-string fiber via the adsorption of nanoparticles, the NPCTi which is the hydrolysate of titanium tetrachloride (TiCl_4_) and the nanoparticles containing Al (NPCAl) which is produced by the hydrolysis of Trimethylaluminium (TMA, Al(CH_3_)_3_). The water collection efficiency of the fibers can be easily controlled via changing not only the size of the beads but also the ratio of the Ti and Al. In addition, we introduce a computational fluid dynamics (CFD) simulation to show the pressure distribution of on the surface of the fibers, which gives another explanation regarding the high water collection efficiency.

## 1. Introduction

Nature always knows what is best for its creations. Multi-scale heterogeneous surfaces and hump-on-string fibers are two of the most common ways of collecting water from the environment [1], such as spider silks [2], cactus spines [3], and even the common bristlegrass [4]. Astonished by the high water collection efficiency of these two structures, a variety of bio-inspired artificial surfaces with multi-scale heterogeneous surfaces and manmade hump-on-string fibers are fabricated and used in collecting water [5,6,7,8], building facilities with super wettability [9,10,11], as well as fabricating phase separation membranes [12]. However, little attention has been paid to the combination of the heterogeneous structures and the hump-on-string fibers.

Here, inspired by the multi-round adsorption of the bead-on-string fibers composed of nanoparticles containing Ti (NPCTi)[13], we fabricated a kind of multi-scale heterogeneous hump-on-string fiber via the adsorption of two kinds of nanoparticles, the NPCTi, the hydrolysate of titanium tetrachloride (TiCl_4_) and the nanoparticles containing Al (NPCAl), generated by the hydrolysis of Trimethylaluminium (TMA, Al(CH_3_)_3_). Also, by adjusting the volume of hydrolyzed TiCl_4_ and TMA, the ratio of Ti and Al composing the humps, as well as the water collection efficiency can be easily controlled.

## 2. Results and Discussion

**Figure 1:**
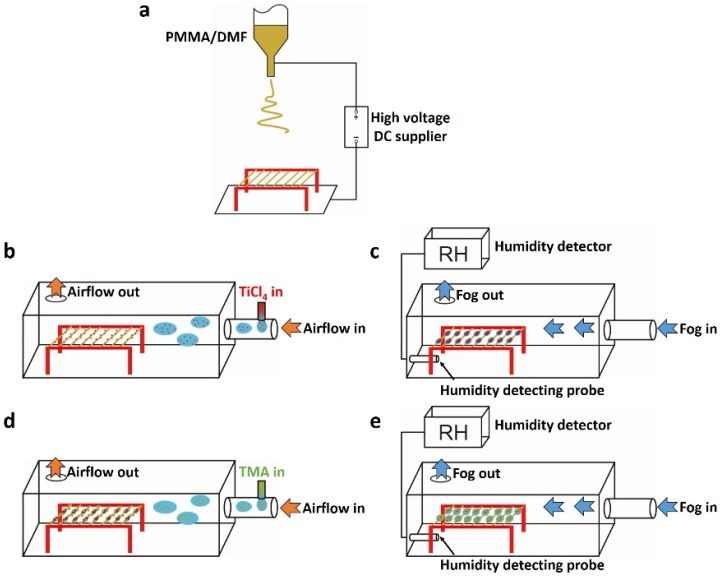
Illustrations of fabrication process of multi-scale heterogeneous hump-on-string fibers (MHHFs). (**a**) Electrospinning process; (**b**) nanoparticles containing Ti (NPCTi) absorption; (**c**) The first wet-assembly process; (**d**) nanoparticles containing Al (NPCAl) absorption; (**e**) The second wet-assembly process.

**Figure 2:**
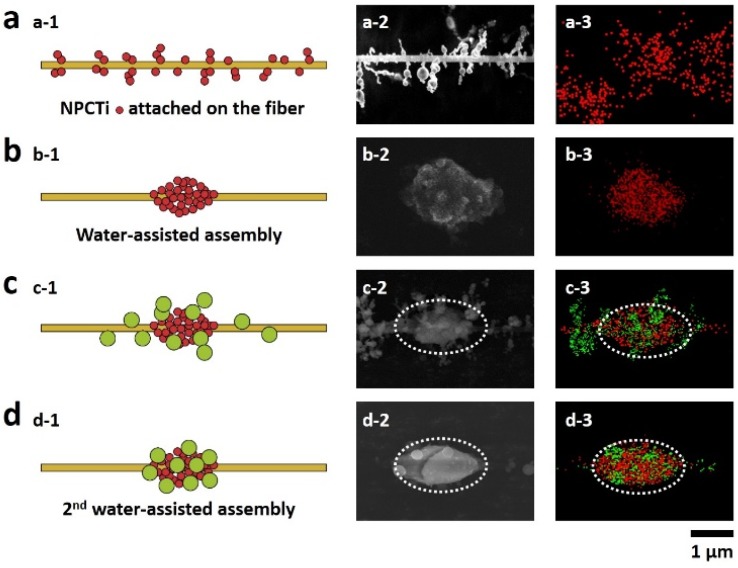
Illustrations and SEM images of the multi-scale heterogeneous hump-on-string fiber. (**a**) NPCTi are adsorbed onto the smooth PMMA fiber; (**b**) the Ti-humps are fabricated after the 1st wet-assisted assembly; (**c**) NPCAl are adsorbed onto the fiber with Ti-humps; (**d**) the multi-scale heterogeneous hump-on-string fibers are formed after the 2nd wet-assisted assembly.

**Figure 3:**
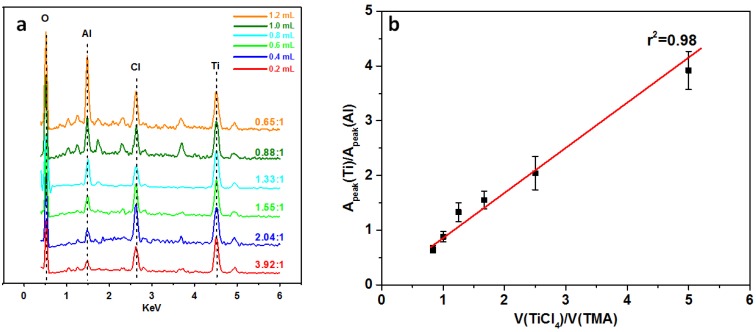
EDS spectrums (**a**) and ratio-control of Ti and Al (**b**) in different fibers (the error bars indicate the standard deviations of the results).

A metal shelf was put under the needle as the collector, on which the parallel PMMA electrospun fibers (0.562 ± 0.035 μm in diameter) can be collected between the two strings (Figure 1a). When the fibers were put into a chamber with two apertures (one for gas in and the other for gas out) and exposed in the smog of the hydrolysis of TiCl_4_, NPCTi, with the average diameter of 0.233 ± 0.054 μm, would be adsorbed onto and randomly surround the fiber, forming a particle-on-string structure (Figure 1b and Figure 2a). By the time the water stream flowed into the chamber from one side and passed through these fibers (the first wet-assembly process), the NPCTi would assemble into highly-ordered micro-humps (Figure 2b, 1.326 ± 0.223 μm in length, 0.885 ± 0.174 μm in width) due to the collection and coalescence of the tiny water droplets. Then, the hump-on-string fibers were put into a similar chamber with apertures with smog caused by hydrolysis process of TMA (Figure 1d). As expected, a series of nanoparticles (Figure 2c, 0.677 ± 0.145 μm in diameter) could be caught by the humps and fibers, which could be identified as NPCAl attaching on the NPCTi-humps via EDS mapping (Figure 2c-3, white oval, Figure S1).

The Ti-hump with a conical shape can cause a difference in the pressure and such a conical shape with a curvature gradient will give rise to a difference in Laplace pressure (Δ*P*) acting on a water drop [2,14]:

(1) $$\text{Δ}P=-\int_{r1}^{r2}\frac{2\gamma }{{\left(r+R_{0}\right)}^{2}}\sin\beta \text{d}z$$

where *γ*, *r*, *R*_0_, *β*, z are the surface tension of the drop, the radius of the fiber, the radius of the spindle, the half apex-angle of the spindle and the direction along the fiber, respectively. In addition, the Ti-hump is more hydrophilic due to its rougher surface and chemical composition and has a higher apparent surface energy than the fiber, Thus, the force generated by a surface energy gradient that arises from a difference in surface roughness and chemical composition can be given by [2,15,16]:

(2) $$F=\int_{L_{f}}^{L_{s}}\gamma \left(\cos\theta_{s}-\cos\theta_{f}\right)\text{d}l$$

where *γ* is the surface tension of water; *θ*_s_ and *θ_f_* are the contact angles of water drop on the spindle and the fiber. According to cos*θ*_w_ = εcos*θ* (ε is the roughness of the surface), *θ*_s_ < *θ_f_*, so *F* has a direction pointing to the spindle. Under the efforts of the collaboration of Δ*P* (geometric gradient) and *F* (chemical gradient), those larger NPCAl can gather around the Ti-humps (the second wet-assembly process), forming the multi-scale heterogeneous hump-on-string fibers (MHHFs, Figure 1e and Figure 2d, 2.136 ± 0.267 μm in length, 1.045 ± 0.155 μm in width).

Considering that MHHFs are fabricated by NPCAl attaching on the NPCTi-humps (Four main peaks appear in the spectrum in Figure 3, which can be seen as the combination of the spectrum of NPCAl (Al peak and O peak) and NPCTi (Ti peak; Cl peak and O peak)); the ratio of Ti and Al can be controlled by adjusting the volume of hydrolyzed TMA and TiCl_4_. When the volume of TMA varies from 0.2 to 1.2 mL while TiCl_4_ is set to 1 mL, the Al peak (E_Kα_ = 1.49 KeV) rises obviously (Ti peak (E_Kα_ = 4.51 KeV) is set to the same height during the characterization); with the ratio of Ti and Al decreasing from 3.92:1 (Figure 3, red line at the bottom) to 0.65:1 (Figure 3, orange line at the top). Besides, a linear correlation is found between (*r*^2^ ≈ 0.98) the ratio of TiCl_4_ and TMA and the ratio of Ti and Al, which may be attributed to the similar process of the adsorption and wet-assisted assembly of NPCTi and NPCAl.

**Figure 4:**
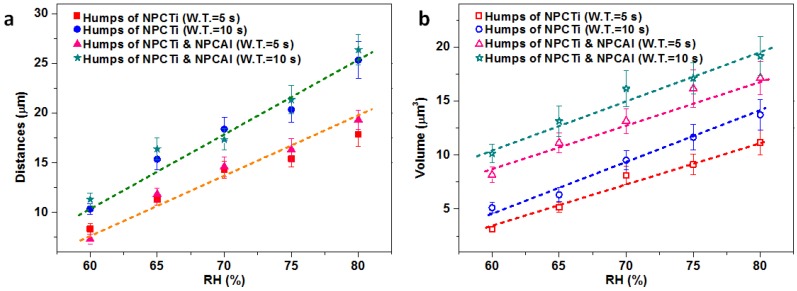
Comparison of distances (**a**) and volumes of humps (**b**) composed of (NPCAl + NPCTi) and NPCTi at different humidity (the error bars indicate the standard deviations of the results).

Based on a similar mechanism of wet-induced assembly, the distances and volumes of the humps on MHHFs can also be adjusted by controlling the humidity of the chamber (noted as RH in Figure 4) and the wetting time (noted as W.T. in Figure 4). As for the distances (Figure 4a), the intervals between humps on MHHFs are from ~5 μm when RH ~ 60% to over 15 μm when RH reaches ~ 80% at a short wetting time (*i.e.*, 5 s) and can be from near 10 to 24 μm with the RH increasing also from ~60% to ~ 80% at a longer wetting time (10 s). Moreover, their trends are nearly the same as those for humps composed of single NPCTi, which may due to the similar collecting-coalescence process of these two kinds of fibers. However, the volumes of the humps are larger than the humps composed of single NPCTi. As RH ~ 60% in wetting time of 5 s, the volumes of humps of NPCTi are only 3.127 ± 0.285 μm^3^ while the volume of humps on MHHFs can be 8.144 ± 0.725 μm^3^. Despite the diversity between the values of volumes, the two kinds of fibers also shared a similar trend line when the RH increases (Figure 4b, the dashes).

**Figure 5:**
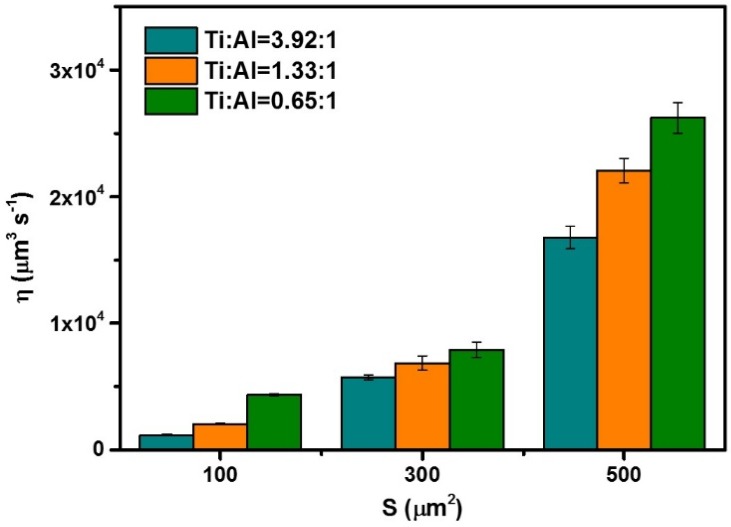
Water collection efficiency of the MHHFs at different ratios of Ti and Al (the error bars indicate the standard deviations of the results).

Compared with fibers with beads composed of single NPCTi, the water collection efficiency of MHHFs is affected by not only the surface area of the beads, but also the ratio of Ti and Al. The results of the water collection efficiency of MHHFs are shown in Figure 5. According to the scaling law of water droplet growth, the efficiency (*η*) can be roughly defined as *η* = d*V*/d*t* = *n*S*V*_M_ [17], which means the small change of the drop volume (d*V*) during a short time period (d*t*) can be estimated by the mole number (*n*) and the molar volume (*V*_M_) of the liquid, as well as the effective collection surface (*S*) of the beads. In this article, we take *η = V*_w_/*t* = 4/3 π (*r*_w_)^3^/*t* (where *V*_w_, *r*_w_ and *t* represent the total volume and radius of the drop and the water collecting time) and S = π (*l*^2^+*α*_w_^2^/tan*α*)/2, where *α* = arcos (*l/w*), *l* and *w* are length and width of bead, respectively. The water collection efficiency of MHHFs increases from ~0.5 × 10^4^ to ~2.5 × 10^4^ μm^3^ when the average collection surface area changes from ~100 to ~500 μm^2^ (Figure 5), which indicates that larger beads have a higher efficiency in water collection than that of smaller ones due to stronger water capturing ability [17,18,19,20,21]. Besides, when the ratio of Ti decreases from 3.92 to 0.65, the water collection efficiency goes up (Figure 5), which may be a result of the chemical and geometric gradient due to the heterogeneous and multi-scale on MHHFs [5].

In order to explain the wet-induced assembly process and water collection property more theoretically, a numerical simulative calculation based on computational fluid dynamics (CFD) and the Navier-Stokes equation (N-S equation) [22] is introduced to simulate the flow field, including the distribution of the pressure and the velocity. As depicted in Figure S2, periodic conditions are taken at the lateral boundaries. The velocity of air is assigned to be 1 m/s at the inlet and a zero diffusion flux for all flow variables is specified at the outflow. Symmetry conditions are employed at the upper and lower boundaries. For the fiber surfaces, no-slip wall boundary conditions are applied, and the wall temperature is assumed to be 298K, which equals to the temperature of the free stream.

**Figure 6:**
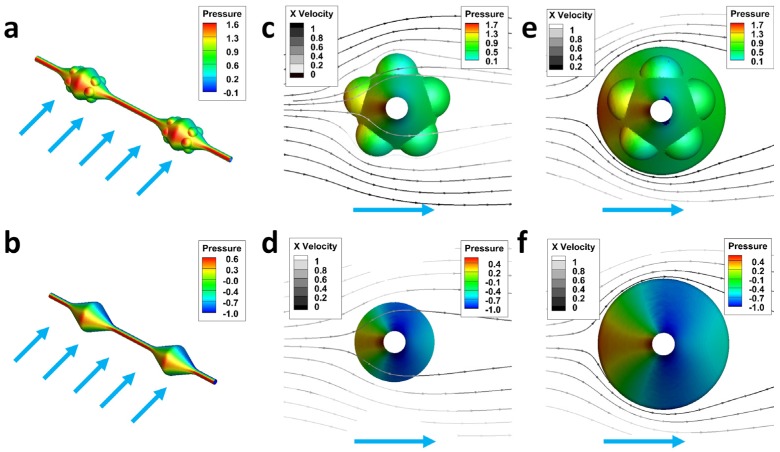
Computational fluid dynamics (CFD) simulation nephograms of bead-on-string fiber with humped and smooth surface. Perspective view of the bead-on-string fibers with humped (**a**) and smooth surface (**b**); Comparison of the side view of the fibers in the humped position (**c**) and the same position on the smooth fiber (**d**); Comparison of the distribution of streamlines at the beads with maximum diameter on the humped (**e**) and smooth (**f**) fiber.

The comparisons of the CFD simulation nephograms between the bead-on-string fiber with humped and smooth surface reveal the reason why the MHHFs have a stronger water collection ability (Figure 6). A larger area with higher pressure occurs on the surface of the bead-on-string fibers with nanoparticles attached (Figure 6a, red zone, *P*_max_ = 1.6 Pa) than the bead-on-string fibers, which have a smooth surface (Figure 6b, *P*_max_ = 0.6 Pa). Such a difference in the distribution in pressure can also be reflected by a more chaotic and denser distribution of streamline. As a result of the blocking and interference effect of the particles, the streamlines have much more curved traces (Figure 6c, grayscale lines with arrows) than those passing through the smooth surface (Figure 6d, grayscale lines with arrows), making both *P*_max_ and *P*_min_ on the surface of humped bead-on-surface fibers higher than those on the fibers with smooth beads at where the particles attached (hump: Figure 6, *P*_max_ = 1.7 Pa, *P*_min_ = 0.1 Pa; smooth: Figure 6d, *P*_max_ = 0.6 Pa, *P*_min_ = −1.0 Pa) and the middle of the beads (hump: Figure 6e, *P*_max_ = 1.7 Pa, *P*_min_ = 0.1 Pa; smooth: Figure 6f, *P*_max_ = 0.6 Pa, *P*_min_ = −1.0 Pa). From the view of diffusion, the Brownian motion becomes intense with decrease of fragment size according to Stokes-Einstein equation [23], which proves that the tiny water drops are much more easily to be caught by the particles than the fiber [18,19,20,21]. 

Here, from another perspective of thermodynamics, according the Kelvin’s Law:

(3) $$\ln\frac{p_{r}}{p_{\infty }}=-\frac{M}{\rho RT}\frac{2\sigma }{r}$$

where *p_r_*, *p*_∞_ represent the pressure of the drops (radius = *r*) and the pressure of the flat surface (radius = ∞); *M*, *ρ*, *R*, *T* are the mole mass, density of the stream, ideal gas constant, and temperature, respectively, which can be seen as constant in this situation. Such a higher pressure field around the MHHFs may be benefit for the water condensation and leads to a stronger ability in water collection.

## 3. Experimental Section

### 3.1. Equipment and Material

The electrospinning process was carried out by NaBond Nanofiber Electrospinning Unit (Nabond Technologies Co., Ltd, Shenzhen, China), a production of Micro and Nano Technology Expert. The magnetic stirrer was purchased from IKA Works (Staufen, Germany). The nebulization process was controlled by Ultrasonic nebulizer NB-150U, which was obtained from Omron Co., Ltd (Dalian, China) (the diameter of nebulizer droplets: 5 μm, according to the technical parameters). Titanium tetrachloride (TiCl_4_) and N, N-dimethyl formamide (DMF) was obtained from Beijing Chemical Works (Beijing, China). Poly-(methyl methacrylate) (PMMA, M_w_ = 300,000) was obtained from Tokyo Chemical Industry Co Ltd (Tokyo, Japan) and trimethylaluminium (TMA, 2.0 M in toluene) was obtained from Aladdin Reagent Co., Ltd (Shanghai, China).

### 3.2. Preparation of PMMA Electrospun Fibers

Exactly 1.5 g PMMA was dissolved into 30 mL DMF to prepare a polymer solution for electrospinning. After being stirred for 2 h at 40 °C and 12 h at room temperature, the polymer solution was loaded into a 10 mL syringe connected by a latex tube (inner diameter of 0.9 mm) with a needle in the end. A unique aluminum shelf was designed as the fiber collector, with its length, width and height of 2.5 cm, 1 cm and 1 cm, respectively. The solution was pumped at a flow rate of 1 mL h^−1^. The distance between the needle and the aluminum shelf was about 20 cm. The voltage was set at 15 kV.

### 3.3. Fabrication of Multi-Scale Beaded Fibers

When the fibers collected on the shelf was exposed to the haze generated by the hydrolysis of the TiCl_4_, nanoparticles of Ti(OH)_n_Cl_(4−n)_ can be caught by the fibers, fabricating the particle-on-string structure. Those nanoparticles can form orderly arranged beads handing in the fibers after the wet-assembly process. TMA, when released in air, can have the similar hydrolysis as TiCl_4_ [24].

Fibers with beads composed with Ti can adsorb the nanoparticles containing Al, forming particle-bead-string structure. After a second wet-assembly process, the nanoparticles containing Al can be gathered on the surface of beads composed with Ti, fabricating the multi-scale heterogeneous beaded fiber.

By changing the volume of TiCl_4_ and TMA injected upon fibers, the ratio of Ti and Al can be easily controlled.

### 3.4. Characterization

Structures of PMMA, PMMA/Ti and PMMA/Ti/Al fibers were observed by scanning electron microscope (SEM, Quanta FEG 250, FEI, Quanta, Hillsbor, OR, USA) at 10 kV. Characteristic element mapping analysis were generated via an X-Max Silicon Drift Detector (Oxford Instruments, Oxford, UK) EDS detector. The wetting process was observed and recorded by a Navitar microscope system (Navitar, Inc, New York, NY, USA) at room temperature.

## 4. Conclusions

We fabricate multi-scale heterogenous fibers with bead-on-string structure (MHHFs) with the help of the wet-induced assembly process of different nanoparticle and achieve the control of the distances and volume of the beads easily by adjusting the RH and wetting time. The water collection efficiency of MHHFs, which has a relationship of the collection surface areas and the ratio between Ti and Al, can also be adjusted by the control the hydrolysis volume of TiCl_4_ and TMA. With the help of the CFD simulation, we indicated the pressure distribution on the surface of the beads with particles attached being higher than those with a smooth surface, which gives an explanation for the high water collection efficiency of the hump-on-string beads. This MHHF is promising for fabricating smart bio-mimetic functional materials that can be applied into the micro-devices, sensor, microfluidics, micro-reactor, water collection and water transport as well.

## Supplementary Materials

Supplementary materials can be accessed at: http://www.mdpi.com/1996-1944/8/7/4249/s1.
