# Supplementary material for: Wet-Induced Fabrication of Heterogeneous Hump-on-String Fibers
Source: Materials (Basel). 2015 Jul 13;8(7):4249–57. doi: 10.3390/ma8074249 (PMC5455660; doi:10.3390/ma8074249)
Supplement: Supplementary file 1 [file materials-08-04249-s001.pdf]

Supplementary Materials

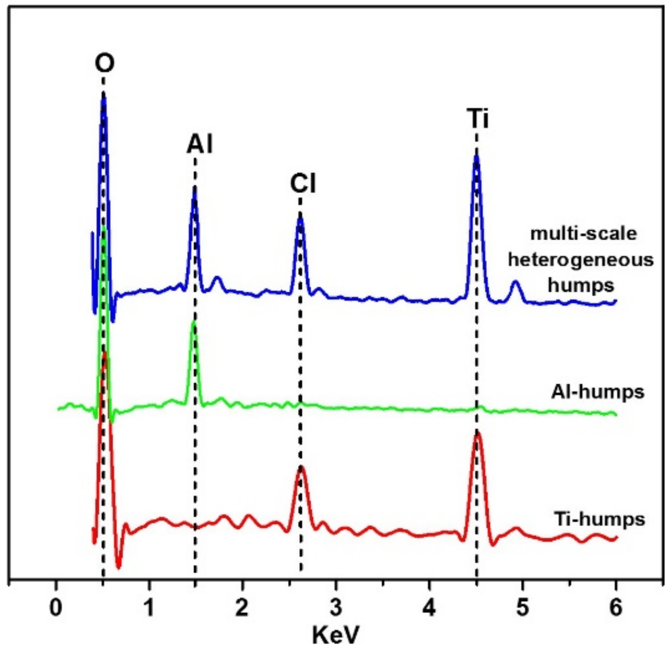

Figure S1. EDS results of Ti-humps; Al-humps and humps on MHHFs.

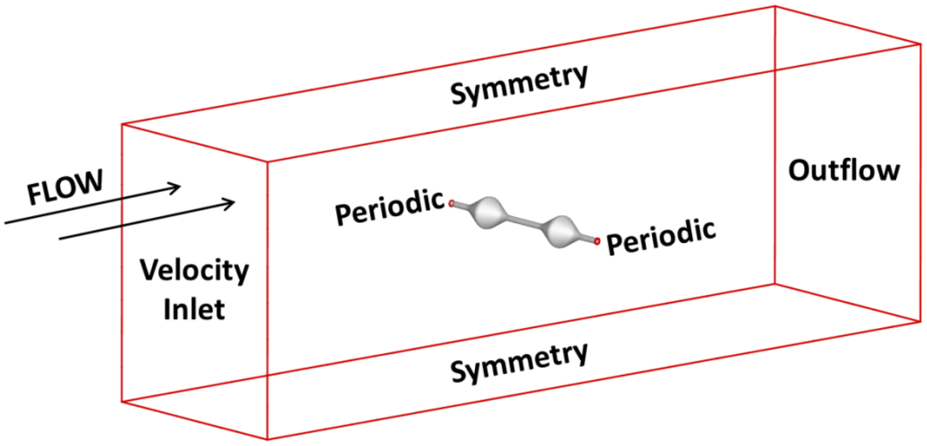

Figure S2. Sketch of computational domain and boundary conditions.
